# Understanding burden of illness for child growth hormone deficiency

**DOI:** 10.1007/s11136-017-1529-1

**Published:** 2017-02-28

**Authors:** Meryl Brod, Suzanne Lessard Alolga, Jane F. Beck, Lars Wilkinson, Lise Højbjerre, Michael Højby Rasmussen

**Affiliations:** 1 The Brod Group, 219 Julia Avenue, Mill Valley, CA 94941 USA; 2 Novo Nordisk A/S, Vandtårnsvej 114, 2860 Søborg, Denmark

**Keywords:** Growth hormone deficiency, Burden of illness, Patient experience, Patient-reported outcomes

## Abstract

**Purpose:**

Research demonstrates that children and adolescents with growth hormone deficiency (GHD) are impacted in multiple ways beyond their short stature; however, there are no disease-specific measures to assess these impacts. The purpose of this study was to examine the burden of GHD on children and adolescents, and to conduct concept elicitation to develop a model of the impact of GHD to support a disease-specific outcome measure.

**Methods:**

Four focus groups and 52 telephone interviews were conducted with children with GHD and parents/guardians of children with GHD to understand the experience and impacts from the child’s perspective, reported by children or parent-observers about the impact on the child. The interviews and focus groups were conducted in Germany, the United Kingdom, and the United States. Interview transcripts were analyzed thematically based on modified grounded theory principles.

**Results:**

There were 73 descriptions of patient’s experiences elicited from 70 respondents, as three respondents spoke for two children each. A majority of GHD descriptive narratives refer to boy children (*n* = 51, 69.9%) and a majority of children had taken GHD treatment (*n* = 64, 89%). Analysis identified four major areas of GHD impact: Signs and Symptoms (beyond short stature), Physical Aspects of Daily Life, Social Well-Being, and Emotional Well-Being.

**Conclusions:**

The burden of GHD in children and adolescents is considerable and not limited to short stature. The severity of GHD impact on children and adolescents appears to be variable and individualized, but these data indicate that early identification and growth hormone treatment may lead to fewer impacts.

## Introduction

Growth hormone deficiency (GHD) results when the pituitary gland does not produce enough growth hormone to stimulate the body to grow and manifest as a slow or flat rate of growth in both early and later childhood [1]. Children with GHD usually have typical body proportions, but are often chubbier, shorter, and may be perceived to be younger than their age when compared with peers of the same age and gender [2, 3]. The prevalence of childhood GHD reported in published studies is within the range 1.8–2.9 per 10,000 in Europe and US [4–6]. A recent study reported the incidence of childhood onset GHD to be 2.58 for males and 1.70 for females per 100,000 [7]. The cause of most childhood onset GHD is not known (idiopathic) [1] or can be genetic or syndromic (developed in utero); it can also develop as the result of an injury or medical condition such as head trauma or brain tumor [1].

Children with GHD may exhibit psychological and behavioral impacts. Although some of these impacts may be directly related to short stature, there are additional proximal impacts which are not directly related to short stature such as academic underachievement due to poor concentration [8]. In fact, improvement in height alone is not necessarily sufficient to improve quality of life (QoL) for GHD children as compared to improvements in QoL for children with idiopathic short stature [9]. Unfortunately, although there are measures of the impact of short stature, no disease-specific measures currently exist which assesses the impact of GHD on children and adolescents. The purpose of this study was to examine the burden of GHD on children and adolescents and to conduct concept elicitation to develop a model of the impact of GHD with adequate conceptual validity to support the development of disease-specific patient-reported outcome (PRO) and observer-reported outcome (ObsRO) measures.

## Methods

Focus groups and interviews with children, ages 8 to <13 years, diagnosed with GHD and parents of children, ages ≥4 to <13 years, diagnosed with GHD were conducted in Germany, the United Kingdom (UK), and the United States (US). Both focus groups and individual interviews are acceptable methodologies to collect qualitative data with children approximately aged 8 or older [10] as well as with adults. However, as it was expected, given the challenges of recruiting children with GHD, that it could be difficult to recruit sufficient numbers of interviewees in any given location for focus groups, the methodology used in each country was determined by the geographic distribution of the respondents. Focus groups conducted with children were divided by gender (one for boys, and one for girls) and for child telephone interviews, the child was asked if they preferred a gender-matched interviewer. A purposive sampling method was used for both focus group and interview selection. This study was conducted from April 2014 to March 2015 and was approved by the Western Institutional Review Board (WIRB tracking #20140677).

Child respondents were eligible if they met the following criteria: (1) pre-pubertal, ages 8 to <13 years with a diagnosis of isolated Growth Hormone (GH) deficiency, GH deficiency as part of multiple pituitary hormone deficiencies, or organic GH deficiency; (2) diagnosed with GHD as defined by a maximum stimulated GH < 10 ng/mL (μg/L) on two separate stimulation tests performed either on the same day or on two separate days, OR one stimulation test performed along with an IGF-1 test resulting in a maximum stimulated GH < 10 ng/mL (μg/L) and an IGF-1 level two standard deviations below the mean reference range for age and gender; (3) negative signs for intracranial tumor or tumor growth, OR if GH deficiency occurred after treatment for any brain tumor, the patient has to be at least one year in clinical remission; (4) currently receiving any prescription GH treatment for no more than 12 months, or never treated with growth hormone. Parent/guardian respondents were eligible if their child met the diagnostic and medical criteria noted above; however, the age of their child could be any age under 13 years. Additionally, parent respondents were required to live in the same residence as the child with GHD and be able to provide information on the child’s GHD and treatment as well as report on the child’s observed behavior.

Child and parent respondents were excluded from the study if the child had (1) any clinically significant abnormality likely to affect growth or the ability to evaluate growth, such as, but not limited to, chronic diseases like renal insufficiency, spinal cord irradiation, and malnutrition; (2) overt diabetes mellitus (fasting blood sugar >126 mg/dl) and impaired fasting sugar (fasting blood sugar >100 mg/dl after repeated blood analysis); (3) chromosomal abnormalities and medical syndromes (Turner’s syndrome, Laron syndrome, Noonan syndrome, or absence of growth hormone receptors), with the exception of septo-optic dysplasia; (4) congenital abnormalities (causing skeletal abnormalities), Russell–Silver syndrome, and skeletal dysplasia.

Three recruitment strategies were employed. First, eligible child and adolescent patients were identified by physicians from their current patient caseload. The physician or designated staff person contacted the parent/guardian of eligible patients to determine their, and/or their child’s, interest in the study. Once permission to share their interest was received, this information was forwarded to the study coordinators who continued the recruitment process. For the second strategy, national and international GHD-related advocacy and support organizations posted recruitment information on their discussion and social media sites, providing contact information for the study for interested respondents. Lastly, respondents were identified by professional research organizations, either through physician referrals or from their patient panels. These organizations contacted individuals enrolled in their proprietary databases and prequalified them by telephone using a screening script. For anyone who was recruited outside of a clinical setting, evidence of diagnosis was obtained by a signed letter from their physician providing confirmation that the respondent met all eligibility requirements. There were target quotas for ages of children. Additionally, a distribution across economic groups, gender, treatment status (naïve vs. on treatment), and length of time on treatment was monitored to ensure representation in the total sample. Participants were compensated USD $125 (or country equivalent) for in-person focus groups or USD $100 (or country equivalent) for telephone interviews as an honorarium regardless of recruitment methodology.

To guide the interviews, a semi-structured interview guide was designed, based on available research and discussion with eight clinical experts in individual concept elicitation interviews (in the US, UK, and Germany), to elicit the perceived symptoms, burden, and impacts of GHD as well as GHD treatment effects on social, physical, and psychological aspects of daily living. Items and probes were designed to be age-specific to accommodate child respondents. Questions in the guide were designed to be both broad, such as “What is the worst thing about having a growth condition and why?” or specific about a particular impact, such as “How much does your growth condition impact your concentration (focus) or your ability to pay attention in and out of school?”. For those children who had been treated for their GHD, questions asking about perceived differences pre- and post-treatment were also included. Further, it was expected that young children (under approximately age 8 years) would not be able to complete a PRO measure by themselves [10] and that a parent ObsRO measure, and not a proxy measure (where the reporter responds to what they think or perceive about the child), would be needed. Therefore, it was critical for the parents to report only actual and not perceived or presumed impacts on the child. In order to facilitate observer rather than proxy reports, parents were instructed to answer questions about what they had themselves actually witnessed or been told about by another person as having witnessed. To ensure this was the case, follow-up questions for impacts reported by parents asked the parents to report concrete examples of what they had seen or been told that lead them to report on the impact.

Trained individuals with backgrounds in qualitative interviewing conducted all interviews, and the interviews were conducted in the native language of the host country. The individual child and parent/guardian telephone interviews were 60–75 min long and the in-person focus groups were two hours long. The interviews and focus groups were recorded, transcribed, and translated into English where appropriate.

Data were qualitatively analyzed through an adapted grounded theory approach, entailing developing and refining a theory based on concepts derived during the research process [11]. Transcripts were analyzed for content by theme using Dedoose, version 7.0.21, a qualitative data analysis system [12]. A preliminary code list was developed based upon the interview and focus group guide, and codes were added as new themes emerged during transcript review. The transcripts were coded in the chronological order in which the interviews and focus groups occurred and were each reviewed and coded at least three times (and often more) to insure accuracy and consistency. These themes were then aggregated into larger domains.

Based on the findings from the qualitative analysis, a conceptual model of the major and minor disease-specific impacts (other than short stature) was developed. To be included in the model, the impact needed to be a discrete rather than a broad descriptor of the impact (e.g., worry rather than general dislike) and could potentially be affected by treatment. Major impacts were those reported by 15% or more of the study sample and a minor impact if reported by less than 10% of the sample. Items that were endorsed between 10 and <15% of the sample were individually examined and included as major if they were endorsed by 20% of parents or children, were proximal rather than distal impacts, not solely related to height, and/or considered conceptually important by clinical experts interviewed (for concepts that clinicians would be aware of) or in the literature. All major impacts were confirmed as both relevant and important by respondents during the cognitive debriefing interviews (conducted after the item generation was completed with an independent sample of parents and children). Additionally, there were three impacts that had 15% or higher endorsement by the sample, which after review and consideration, were categorized as minor. For example, “reaching things” was not perceived as a burden as adaptations were readily available.

## Results

### Sample description

Thirty-nine children and adolescents ages 8 to <13 years with GHD and 31 parents of children ages 4 to <13 years with GHD participated totaling 70 respondents. Fifty respondents (71.4%) were recruited directly from clinical contacts, 4 (5.7%) from professional research organization patient panels, and 16 (22.9%) from advocacy and support organizations. Seven children were aged 8–9 years and 32 children were aged 10 to <13 years. Of the 31 parents, 19 were parents of child respondents who participated in the study. Parent respondents provided descriptions of 14 children between the ages of 4 and <8 years, 8 children ages 8–9 years, and 12 children ages 10 to <13 years. All data collected in Germany were by focus group (4 focus groups, 19 respondents). However, the broad geographic distribution of eligible respondents in the UK and US made focus groups logistically unfeasible; thus, all data in these countries were collected from telephone interviews (51 individual interviews). Three parents were interviewed about two of their children with GHD (two in Germany, one in the US). Therefore, for analytic purposes, there was an *N* = 73 for the number of narrative descriptions of GHD.

Of the GHD descriptions gathered by interview or focus group, slightly over half were from respondents in the US (*n* = 40, 54.8%) with additional respondents from Germany (*n* = 21, 28.8%) and the UK (*n* = 12, 16.4%). Table 1 presents the breakdown of respondents by country and age of child.

**Table 1:** Summary of parent and child participants by country and age (years) of child

Country	Child age 4 to <8		Child age 8–9		Child age 10 to <13		#GHD Descriptions (*N* = 73); # (%)
	*Child*	*Parent*	*Child*	*Parent*	*Child*	*Parent*	
Germany	–	6	4	4	7	–	21 (28.8)
United Kingdom	–	4	1	1	6	–	12 (16.4)
United States	–	4	2	3	19	12	40 (54.8)
TOTALS	–	14	7	8	32	12	73 (100)

The mean age of GHD diagnosis was 7 years (range 3–12 years) for the entire sample; however, the average age of diagnosis was the lowest in Germany (4.6 years) as compared to the UK (7.0 years) and US (9.4 years). A majority of children had taken GH therapy (*n* = 66, 90.4%) with treatment beginning on average in Germany at a younger age (4.9 years) than in the UK (7.9 years) or US (9.5 years) due to this earlier age of diagnosis. As a result, the average length of time on treatment varied considerably with the longest time on treatment reported in Germany (43.0 months), followed by the UK (7.9 months) and US (6.5 months). Respondents reported many additional health conditions; the most frequent were ear, nose, and throat (ENT) conditions; lung diseases or other respiratory conditions (including asthma and allergies); and mental health conditions (including depression and anxiety). Table 2 presents the details on the health and demographic characteristics of children and parents associated with the 73 narrative descriptions.

**Table 2:** Health and demographic characteristics of children with GHD described in the study

Demographic characteristics	Germany (*n* = 21)	UK (*n* = 12)	US (*n* = 40)	Totals (*N* = 73)
Age group counts, # (%)				
Age 4 to <8 years	6 (28.6)	4 (33.3)	4 (10.0)	14 (19.2)
Age 8–9 years	8 (38.1)	2 (16.7)	5 (12.5)	15 (20.5)
Age 10–12 years	7 (33.3)	6 (50.0)	31 (77.5)	44 (60.3)
Gender, # (%)				
Female	8 (38.1)	4 (33.3)	10 (25.0)	22 (30.1)
Male	13 (61.9)	8 (66.7)	30 (75.0)	51 (69.9)
Ethnicity, *n* = 52; # (%)				
White	Not collected^a^	10 (83.3)	36 (90.0)	46 (88.5)^a^
Persian		1 (8.3)		1 (1.9)^a^
Asian		1 (8.3)		1 (1.9)^a^
Other			4 (10.0)	4 (7.7)^a^
Household Income				
Less than USD 20,000	2 (9.5)	1 (8.3)	1 (2.5)	4 (5.5)
USD 20,001–40,000	2 (9.5)	3 (25.0)	2 (5.0)	7 (9.6)
USD 40,001–60,000	1 (4.8)	3 (25.0)	5 (12.5)	9 (12.3)
USD 60,001–80,000	2 (9.5)	1 (8.3)	6 (15.0)	9 (12.3)
USD 80,001–100,000	4 (19.0)		13 (32.5)	17 (23.3)
More than USD 100,000	3 (14.3)	1 (8.3)	9 (22.5)	13 (17.8)
Decline to answer	7 (33.3)	3 (25.0)	4 (10.0)	14 (19.2)
Other prescription medications				
Yes, # (%)	4 (19.0)	2 (16.7)	23 (57.5)	29 (39.7)
Age at diagnosis				
Mean (range)	4.56 (3–8)	7.01 (3–12)	9.36 (3–12)	6.98 (3–12)
Ever taken GHD therapy				
Yes, # (%)	20 (95.2)	11 (91.7)	35 (87.5)	66 (90.4)
Age first started GHD therapy				
Mean (range)	4.87 (4–8)	7.89 (8–12)	9.47 (3–12)	7.41 (3–12)
Duration (months) of GHD Therapy				
Mean (range)	43.0 (1–96)	7.9 (2–12)	6.5 (0.2–16)	17.8 (0.2–96)
Other health conditions				
Arthritis, rheumatic diseases, musculoskeletal conditions			1 (2.5)	1 (1.4)
Ear, nose, and throat conditions	4 (19.0)		6 (15.0)	10 (13.7)
Eye disorders	1 (4.8)	1 (8.3)		2 (2.7)
Kidney disease, urinary conditions		1 (8.3)	1 (2.5)	2 (2.7)
Lung disease, respiratory conditions (including allergies and asthma)	2 (9.5)		15 (37.5)	17 (23.3)
Mental health conditions (including depression and anxiety)			13 (32.5)	13 (17.8)
Metabolic conditions (including elevated cholesterol)	2 (9.5)	1 (8.3)	1 (2.5)	4 (5.5)
Stomach, intestinal, gastrointestinal disease		1 (8.3)		1 (1.4)
Stroke, neurological condition		1 (8.3)	2 (5.0)	3 (4.1)
Other condition	1 (4.8)	4 (33.3)	4 (10.0)	9 (12.3)
None	13 (61.9)	6 (50.0)	15 (37.5)	34 (46.6)

Ethnic identifications were not collected in Germany due to ethics standards in Germany

^a^ *n* = 52

### Domains and themes generated by telephone interviews and focus groups

A total of 71 concepts emerged from all interviews and focus groups conducted; 54 of these concepts were addressed by participants in both the child and parent/guardian samples. Thematic saturation was separately assessed for the 39 children and 34 parents/guardians in the order in which the interview or focus group occurred. A total of 55 concepts were discussed during the child interviews; after the 18th child interview, 80% of these concepts had been discussed, and by the 34th interview, 95% of these concepts had been covered. A total of 70 concepts were discussed during the parent/guardian interviews; after the 9th parent/guardian interview, 80% of these concepts had been discussed, and by the 20th interview, 95% of these concepts had been covered. The four disease impact domains identified were Signs and Symptoms, Physical Aspects of Daily Life, Social Well-being, and Emotional Well-being. The descriptions of subthemes by domain are as follows.

#### Signs and symptoms

Parent and child-provided descriptions of GHD suggest that the subjective experience of GHD is variable and individualized. Respondents described a range of symptoms in addition to short stature (*n* = 38, 52%) that lead to additional impacts in daily, emotional, and social aspects of life. Signs and symptoms reported included poor appetite (*n* = 34, 47%), reduced strength or poor muscle development (*n* = 34, 47%), poor energy levels (*n* = 25, 34%), reduced endurance (*n* = 22, 30%), poor sleep (*n* = 22, 30%), poor focus or concentration (*n* = 13, 18%), and fatigue (*n* = 11, 15%). Parent and child respondents were in close agreement about the two most frequently reported symptoms; parents reported poor appetite (*n* = 22, 65%), reduced strength (*n* = 17, 50%), and reduced endurance (*n* = 14, 41%), while children reported reduced strength (*n* = 17, 44%), poor appetite (*n* = 12, 31%), poor energy (*n* = 12, 31%), and poor sleep (*n* = 12, 31%). The variety of symptoms and the frequency that respondents denied those symptoms they did not experience suggest that symptom distribution is variable. For example, approximately one-third reported a problem with poor energy (*n* = 25, 34%), but approximately one-quarter reported poor energy was not a problem for them when asked (*n* = 17, 23%). However, only a very few commented that there were no symptoms associated with GHD other than small stature (*n* = 3, 4%), suggesting that most children with GHD experience at least some symptoms. Key modifiers for these symptoms include age of GH treatment initiation and duration of treatment with children starting treatment earlier or having been on treatment longer having fewer impacts.

There were little to no differences in the narrative descriptions of signs and symptoms for boy and girl children with GHD. However, there were some distinctions in reporting by age group. Symptoms were noted at higher frequency for children 4 to <8 years including poor appetite, poor strength or muscle development, poor energy, reduced endurance, poor focus, and fatigue. Table 3 presents the breakdown of all subthemes within the Signs and Symptoms domain.

**Table 3:** Sign and Symptoms domain by subtheme

Sign and Symptoms domain	Total narrative descriptions		Child-provided descriptions		Parent-provided descriptions	
	*N* = 73	%	*N* = 39	%	*N* = 34	%
Smaller or smallest among peers	38	52	13	33	25	74
Poor appetite	34	47	12	31	22	65
No problem with appetite	3	4	0	0	3	9
Reduced strength/poor muscle development	34	47	17	44	17	50
No problem with strength	6	8	0	0	6	18
Poor energy	25	34	12	31	13	38
No problem with energy	17	23	7	18	10	29
Reduced endurance	22	30	8	21	14	41
Poor sleep	22	30	12	31	10	29
Poor focus or concentration	13	18	5	13	8	24
No problem with focus	15	21	10	26	5	15
Fatigue or tiredness	11	15	3	8	8	24
Impaired immune system	4	5	1	3	3	9
No symptoms noted	3	4	0	0	3	9

#### Physical aspects of daily life

Parent and child-provided descriptions of GHD indicate that there are several aspects of daily life impacted by symptoms of GHD. The overarching theme was the level of physical activity, which included reduced performance in physical activities/sports (*n* = 43, 59%), reaching things (*n* = 32, 44%), and limits of what they could do because of size (*n* = 14, 19%) (Table 4). Additionally, these impacts were often described as improving with GHD treatment. Parent and child respondents were in agreement about the two biggest impacts: reduced performance in physical activities or sports (parent *n* = 23, 68% and child *n* = 20, 51%) and reaching things (parent *n* = 16, 47% and child *n* = 16, 41%). However, of note, reaching things was not perceived as a burden as adaptations were readily available. None of the respondents commented that there were no daily life physical impacts for children with GHD. Key modifiers for these impacts include age of treatment initiation and duration of treatment. Children who started treatment at a younger age or had been on treatment longer had fewer physical impacts.

**Table 4:** Physical aspects of daily life domain by subtheme

Physical aspects of daily life domain	Total narrative descriptions		Child-provided descriptions		Parent-provided descriptions	
	*N* = 73	%	*N* = 39	%	*N* = 34	%
Reduced performance in physical activities/sports	43	59	20	51	23	68
No problem with physical activities/sports	18	25	14	36	4	12
Reaching things	32	44	16	41	16	47
No problem reaching things	5	7	4	10	1	3
Limits/not allowed to do things because of size	14	19	5	13	9	26
Low Weight or Underweight	11	15	3	8	8	24
Overweight	3	4	1	3	2	6
Additional difficulty with daily activities	9	12	1	3	8	24
Difficulty with climbing stairs	8	11	1	3	7	21
Difficulty with toileting	5	7	0	0	5	15
Needs booster seats longer	1	1	0	0	1	3
Delayed puberty	2	3	0	0	2	6

Descriptions of girl children with GHD included more frequent reports of reduced performance in physical activities or sports. Parent-provided descriptions of children between the ages of 4 and <8 years emphasized difficulty reaching things with greater frequency than the 8–9 years or 10 to <13 years age groups.

#### Social Well-Being

The social impacts of GHD reported by the respondents were often a result of the child’s visibly smaller size and often contributed to the emotional impacts of the condition. These included being mistaken for a younger age (*n* = 41, 56%), teasing or bullying (*n* = 35, 48%), being treated differently than peers by adults (*n* = 20, 27%) or other children (*n* = 20, 27%), social unease or not fitting in (*n* = 19, 26%), choosing not to participate or holding back from participation (*n* = 18, 25%), and exclusion from activities/playing (*n* = 7, 10%) (Table 5). For many of the respondents, it was the repetition of a negative social experience that contributed to emotional impacts. The most frequent concern for many parent and child respondents was the consistent pattern of being mistaken for younger and feeling underestimated, which resulted in differential treatment by adults and other children. Similarly, to previous impact domains, there was variability in these experiences. Approximately one-fifth of the sample (*n* = 16, 22%) reported that there were no social impacts associated with GHD. An important mediating influence that modulated the negative social experience was social support from family, friends, or teachers and school systems. Social support was reported by approximately a quarter of the sample (*n* = 19, 26%). And although verbal teasing was a strong concern, many reported that they or their child had not endured teasing (*n* = 30, 41%) and some felt that children with GHD were not treated differently by other children (*n* = 13, 18%).

**Table 5:** Social Well-Being domain by subtheme

Social Well-Being Domain	Total narrative descriptions		Child-provided descriptions		Parent-provided descriptions	
	*N* = 73	%	*N* = 39	%	*N* = 34	%
Being mistaken for younger	41	56	17	44	24	71
Teasing or bullying	35	48	14	36	21	62
Teasing but it feels okay	4	5	2	5	2	6
No experience with teasing	30	41	24	62	6	18
No experience with bullying	6	8	6	15	0	0
Treated differently than peers by adults	20	27	4	10	16	47
Treated differently by other children	20	27	5	13	13	38
Not treated differently by other children	13	18	9	23	4	12
Social unease/Not fitting in	19	26	6	15	13	38
No problem with social unease	5	7	1	3	4	12
Experiences social support	19	26	8	21	11	32
Family support	14	19	7	18	7	21
Friend support	5	7	5	13	0	0
Teacher/school support	4	5	0	0	4	12
Clothing/wearing clothes typically worn by younger children	19	26	4	10	15	44
Choosing not to participate/holding back or withdrawing from participation	18	25	4	10	14	41
No problem with participation	6	8	4	10	2	6
No social impact	16	22	8	21	8	24
Tension between siblings due to sibling sizes	10	14	3	8	7	21
No problem between siblings	5	7	4	10	1	3
School Impacts	10	14	3	8	7	21
Missed school time/medical	8	11	2	5	6	18
Held back a year	2	3	1	3	1	3
Excluded from activities/playing	7	10	0	0	7	21
No problem with exclusion	7	10	4	10	3	9
Does not tell others about GHD	7	10	5	13	2	6
Prefers to play with younger children	6	8	1	3	5	15

Although there was little difference between boy and girl children around the theme of verbal teasing, all the descriptions of bullying, defined as having a physical aspect of pushing or fighting, were reported for boy children. Boys were also more frequently described as having social unease or difficulty fitting in with their peers. However, boys were more frequently described as experiencing social support than girls with GHD. Finally, girls were more frequently reported as being treated differently by other children and having clothing size and age appropriateness of clothing concerns.

There were some distinctions in reporting by age group. Verbal teasing, being treated differently than peers by adults and other children, choosing not to participate or withdrawal from activities, and concern about clothing size or age appropriateness of clothing were noted at higher frequency for children between the ages of 4 to <8 years.

#### Emotional Well-Being

Parents and children identified a number of emotional impacts, including generally disliking or feeling bothered by their own height (*n* = 46, 63%) and worry (*n* = 36, 49%) (Table 6). Children with GHD were reported to worry about being or feeling different (*n* = 28, 38%), worry about growing (*n* = 15, 21%), or worry about how they will be treated socially (*n* = 7, 10%). Child and adult respondents also reported poor self-confidence (*n* = 24, 33%), sad or hurt feelings (*n* = 15, 21%), embarrassment (*n* = 13, 18%), anger (*n* = 13, 18%), annoyance (*n* = 13, 18%), frustration (*n* = 11, 15%), feeling upset (*n* = 10, 14%), and feeling nervous or anxious (*n* = 9, 12%). Approximately one-fifth of the sample (*n* = 15, 21%) reported that there were no emotional impacts associated with GHD.

**Table 6:** Emotional Well-Being domain by subtheme

Emotional Well-Being domain	Total narrative descriptions		Child-provided descriptions		Parent-provided descriptions	
	*N* = 73	%	*N* = 39	%	*N* = 34	%
Dislikes/is bothered by height or size	46	63	25	64	21	62
Is not bothered by height or size	25	34	11	28	14	41
Worry	36	49	14	36	22	65
Worries about being/feeling different	28	38	9	23	19	56
Worries about growing	15	21	6	15	9	26
Worries about social treatment	7	10	5	13	2	6
Poor self-confidence	24	33	5	13	19	56
No problem with self-confidence	8	11	0	0	8	24
Sad or hurt feelings	15	21	7	18	8	24
No emotional impact	15	21	6	15	9	26
Embarrassment	13	18	7	18	6	18
No problem with embarrassment	18	25	12	31	6	18
Anger	13	18	2	5	11	32
No problem with anger	2	3	1	3	1	3
Annoyance	13	18	4	10	9	26
Compensation strategies for small size	13	18	0	0	13	38
Big personality	11	15	0	0	11	32
Verbal strategies/negotiation	2	3	0	0	2	6
Frustration	11	15	2	5	9	26
Upset	10	14	2	5	8	24
Nervous or anxious	9	12	2	5	7	21
Poor self-image	5	7	1	3	4	12
Positive emotional impacts						
Perceives some benefit to small size	17	23	13	33	3	9
Likes being small in size	12	16	8	21	4	12
Acceptance of GHD	12	16	8	21	4	12
Emotional impacts on Parents						
Worry	16	22	0	0	16	47
Anger or frustration	13	18	0	0	13	38
Relief or content when a diagnosis was made	10	14	0	0	10	29
Pressure or tension, feeling stressed	4	5	0	0	4	12

Many of the emotional impacts described resulted from social impacts of GHD or being bothered by their height or size. Child and parent respondents reported most frequently on the same emotional impacts but, not surprisingly, children were less willing or able to report these negative emotional impacts than were parent respondents. For example, worries about being/feeling different were reported by 9 (23%) children and 19 (56%) parents and poor self-confidence by 5 (13%) children and 19 (56%) parents. Having a sibling or other family members with GHD was an important modifier for emotional impacts, as was the experience of positive social support from family, friends, and peers.

There were positive emotional impacts reported as well. Many of the child respondents were asked if they could identify benefits to being small and approximately one-quarter of the total sample (*n* = 17, 23% total sample and n = 13, 33% of the child respondents) could report some benefits. These included the ability to fit into small spaces, lower fees for activities because they looked to be a younger age, and the agility to move faster or more flexibly than others. Some child and parent respondents reported that the child liked being small in size or stature (*n* = 12, 16%) and some reported that the child had accepted the diagnosis of GHD (*n* = 12, 16%).

Finally, parents reported that they experienced emotional impacts from their child’s GHD. Among parents, nearly half reported worry for their child (*n* = 16, 47%), anger or frustration over the reactions of others about their child’s size (*n* = 13, 38%), relief or contentment when a diagnosis was made (*n* = 10, 29%), and pressure or tension in parenting and managing treatment for their children (*n* = 4, 12%).

There were some distinctions in reporting by age group. Dislike or feeling bothered by one’s height, worry in general and worry specifically about growth or growing was noted at higher frequency for boys than for girls. Feeling hurt or upset, annoyance, and frustration were noted at higher frequency for girls than for boys. Worry about being different from others and frustration were reported at higher frequency for children between the ages 4 and <8 years.

#### Conceptual and theoretical model

**Fig. 1:**
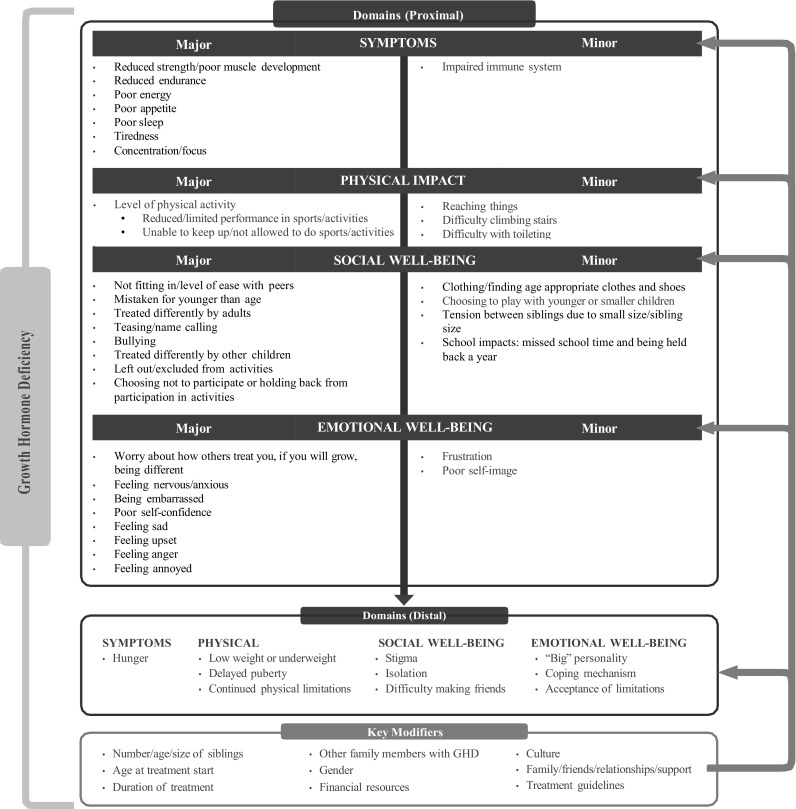
Preliminary disease burden of CGHD theoretical model

The model presents the major and minor proximal and distal impacts of GHD along with key modifiers that can impact the individual experience of the disease (Fig. 1). For a major domain to be included in the model, it had to be endorsed by both children and parents as important and relevant. Table 7 presents the parent and child quotes for each major impact.

**Table 7:** Selected Quotes for Major Themes

Theme/subtheme	Selected quote
*Signs and Symptoms*	
Poor appetite	*Parent: Her appetite has doubled, so now she takes seconds and thirds at dinner and eats quickly, is hungry when she sits down. That aspect of her is a whole new child* *Child: I’m eating more. I used to not be eating all of my plate, but now I’m eating most of it*
Reduced strength or poor muscle development	*Parent: In swimming she was lacking the ability to keep up with her peers in her age group because she was small, her muscle tone was small* *Child: Like a lot of kids could like—because they were tall ones, they were a little bit stronger than me and they could do more things like more pull-ups than me, but I’ve been getting stronger*
Poor energy and/or reduced endurance	*Parent: The biggest impact for him…he isn’t as powerful as others. He is ambitious, but he is tired very fast when he is doing sports. He isn’t as powerful* *Child: Well, before I started the treatment I couldn’t really like run as fast as I am now. And it was like I ran out of breath in short periods of time, rather than keeping—keep running when I was in breath when I was out of breath*
Poor sleep	*Parent: She has never been a good sleeper, but she used to wake up early in the morning, couldn’t go back to sleep, had circles under her eyes* *Child: Yes, I used to never sleep good. Like I used to wake up in the middle of the night and now I sleep better*
Poor focus/concentration	*Parent: Now she can actually sit through a board game or an arts and crafts kind of thing* *Child: When I’m sitting in school and we’re reviewing a test, I’m more focused and I really want to correct everything and really concentrate*
Fatigue or tiredness	*Parent: He sometimes just lay down, because he was totally exhausted after a few hours without having done something special. He didn’t do any sports or ride a bike or whatever, but he just got up, had breakfast, went to the kindergarten, and was totally exhausted by that* *Child: I just like, sometimes just can’t stay awake. I get really tired sometimes*
*Physical aspects of daily life*	
Reduced performance in physical activities/sports	*Parent: Because the year before, we’d noticed – I mean he virtually came last in everything whereas this year when he did his sports day, then he was up in the middle of the pack* *Child: I mean, it would be really hard to, like if we were playing tag, I would feel myself like I wouldn’t run as fast as I really thought I wanted to, and it really bummed me out because I never wanted to play. I mean I played, but it wasn’t really all that enjoyable*
Limits/not allowed to do things because of size	*Parent: I purposely didn’t let him play basketball because of his height* *Child: Like before that when I was younger, I was not able to ride all of the rides. I never went to the amusement park with some of my friends*
*Social Well-Being*	
Being mistaken for younger	*Parent: You know mostly if we go out to a restaurant they always want to give him the kids menu. You know I don’t want the kids menu… He’ll say, “No thank you” or “may I have a regular menu”, but I know it bothers him. He says he doesn’t like it, and his older brother went through it too* *Child: Like everybody would ask who’s older and I would always say, “Me.” Then they would say, “Is she taller?” Yeah, because she looks older since she’s taller than me… At first I didn’t mind, but then everywhere they started asking. Then it started bugging me. And then I was just tired of it*
Teasing or bullying	*Parent: Yeah, well he has a tendency, he keeps things a little bit inside, so he’ll come home from school in a rotten mood, yelling, crying, and you have no idea why and sometimes it’s at bedtime or sometimes it’s a week later that I find out what happened and somebody said something. You’re too little to play this or somebody made a comment about this or… But if he gets upset, that’s kind of like he won’t necessarily tell you right then and there* *Child: I was walking to the bus stop yesterday and then this boy that I didn’t even know that was in a car rolled down his window and shouted “you midget!”*
Treated differently than peers by adults	*Parent: That she was constantly confronted with the fact that people thought that she was younger. Her physical and mental abilities did not match the way she looked like. She was constantly confronted with that discrepancy* *Child: Well they’d talk to me like, hmm… or they would talk to me like if I were nine or younger like using not so big words*
Treated differently by other children	*Parent: I’ve observed some of the kids on his baseball team you know patting him on the head and doing that sort of thing* *Child: People who have just met me, treat me as like I’m younger than… Like if they’re not in the same age as me and I know that and they don’t know that - - they typically don’t know that I’m the same age as them just because I’m shorter than them*
Social unease/not fitting in	*Parent: I knew he just felt very, very uncomfortable in those social situations, and I - - and I guess at that time I didn’t realize it was that strongly tied to his height, but now looking back, it absolutely was. Absolutely that was the whole - - that was the whole reason, there was no other reason* *Child: I used to be - - I think it - - I used to be like always like the one that wasn’t fit in with everyone else, and I think the growth hormone helps me a lot with that one*
Choosing not to participate/withdrawing or holding back	*Parent: Sometimes when other people start off running, you see he’ll run just a couple paces and then he’ll stop. This… Say, “Oh, well, I can’t keep up with them.”* *Child: Like I don’t want to join in because that’s the part where I’m scared most people will make fun of me*
Left out/excluded from activities	*Parent: My son is not accepted by other children of the same age as being a play pal for them. […] The kindergarten people told me that he doesn’t play with them at all because they don’t see him* *Parent: My son started to suffer from the fact that he was so short. The older children didn’t want to play with him anymore. They didn’t want to play with the little ones. And he couldn’t play with the real little ones because the age gap was too big. […] It was difficult for him*
*Emotional Well-Being*	
Worry	*Parent: My son was already worried. He is seven now, and during the last six months he was really worried why he didn’t grow anymore* *Child: I was always smaller than the other kids, but like last year I wasn’t growing at all and I was getting kind of worried*
Poor self-confidence	*Parent: He never said anything specifically about, oh I’m short and boo-hoo, woe is me, but it was just, you just knew that’s what it was. I can’t even explain. I can’t even explain how you would know that, but as a parent you can just tell that he had no - - why would you have no confidence in yourself? People who have no confidence in themselves are you know people who have something that makes them feel uncomfortable about themselves… you can just see it in a child. They behave in a different manner. They’re quiet; they’re not very social, and you know he was definitely that kid* *Child: Like I didn’t feel like good about myself. Like I would always be short for the rest of my life, but I’m feeling better about myself and stuff - like feeling better about myself*
Sad or hurt feelings	*Parent: Lisa sat in the corner, crying, because somebody had said: “hey, little one”* *Child: Could be a little sad because I don’t like want to be short; like you know I don’t want to have that condition at all, yeah*
Embarrassment	*Parent: So I mean that’s just, like you could just see it in his face. Like that was like a slap in the face. You might as well have just punched him, because that’s how he felt. You know he just had to stand there while everybody else was just doing that one, because he couldn’t do it. And I was like - - I came home and I said to my husband my God we got to get him out of that class. You know. Now he doesn’t want to do it anymore* *Child [referring to size]: I just felt embarrassed*
Anger	*Parent: Yeah, even last summer. There was still one ride he couldn’t ride. And we didn’t even talk about it. He would get so mad* *Child: I feel kind of sad and mad my body doesn’t work right, but I’m kind of happy that I can grow more than I would have if I didn’t do it*
Annoyance	*Parent: I would hear people say, “oh you have such pretty eyes, who’s older”, and then Julia would always say “I am”, and then the look that people would give her and then say “but she’s taller”. I would see Julia just roll her eyes like oh one more time, another time, really again* *Child: It gets on my nerves when people say that I am so small*
Upset	*Parent: So he’ll come home from school in a rotten mood, yelling, crying, and you have no idea why and sometimes it’s at bedtime or sometimes it’s a week later that I find out what happened and somebody said something, “you’re too little to play this” or somebody made a comment* *Child: They just keep calling me names and I just want it to stop*
Nervous/anxious	*Parent: I think he’s nervous that he’s going to get teased, so he acts in this different way to sort of head it off at the pass* *Child: I was pretty nervous because I had never really heard of this. I mean one day the doctor just showed up and said you’re below average growth and we’re going to take you to this doctor to look at you. […] The first day I went to the doctor, I was pretty nervous*

## Discussion

This qualitative study provides evidence that the experience of GHD is multi-faceted and that it impacts multiple aspects of daily life for children with the condition. With few exceptions, the experiences are negative and result in substantial disease burden. Their symptoms span a wide range and are often variable and individualized beyond the most basic effect of GHD: small stature and reduced growth. Signs and symptoms limit physical aspects of daily life, thus making them even more visible as different from their peers. This is noticed by their peers and by adults as well. It is apparent that this visibility may lead to further difficulties, resulting in differential social treatment that contributes either as a proximal impact or as a more distal negative emotional impact. As there are many causes of GHD, including prior illness or concussions in addition to genetic susceptibility, it is not surprising that this variability of experience exists for these children.

Some of the physical impacts of daily life such as “reaching up to get things,” social impacts such as “people thinking you are younger than you are,” and emotional impacts such as “worried about growing” link directly to the small stature associated with GHD, its related symptoms, and the inability for children with GHD to reach physical developmental milestones expected of children their age. With growth hormone replacement treatment, many children with GHD can grow to normal height [3] and improvement in self-esteem, emotional well-being, and mood in children with GHD have been shown [13, 14]. If left untreated, or if GHD develops late in childhood, the condition can contribute to shorter-than-average height and delayed puberty [3, 15]. Thus, beginning treatment at an early age could be an important modifier in the experience of GHD and its impacts by facilitating growth and physical ability.

This study also has clinical implications for physicians and their patients. Understanding the considerable impact of GHD on children can improve communications between parents, their children, and physicians. Through an understanding of the consequences of GHD, physicians and parents can better support children with the condition, advocating not only for early treatment but also for a reduction in the social circumstances of teasing, bullying, and exclusion that cause so much harm.

All impacts were found to be relevant to both younger and older children; however, some differences in level of endorsement of impacts for older versus younger children were found. The difference in level of endorsement may have been influenced by the fact that there were no children under the age of 8 who were interviewed and provided self-report, and therefore, impacts reported for younger children were solely reliant upon parents’ observations. Future quantitative research would be helpful to further examine this issue. Finally, the child focus groups in Germany seemed to have a strong cohort effect with little diversity in opinion or experience. While it may be true that the experience of GHD occurs uniformly across these individuals, it may also be true that children, when gathered in groups, tend towards agreement rather than disagreement with one another and that the individual interviews conducted in the United Kingdom and United States were more able to elicit a wider range of experience.

As with all research, there are some limitations to this study. First, although this study included reports from 73 child and parent-provided descriptions of GHD experience (a large sample size for qualitative research), these findings still may not be generalizable to all children with GHD in other countries or ethnic groups. Further, it is possible that volunteers for participation in research form a unique population that results in a sample that may not be representative of all children with GHD or their parents. However, given that recruitment employed three different methodologies, the sample pool was potentially broadened. Finally, quantitative research that investigates the relationship between early treatment and reduced impacts is needed.

## Conclusion

This study demonstrates that the burden of GHD in children and adolescents is considerable and not limited to short stature. GHD includes multiple symptoms for some patients, including poor appetite, poor strength or muscle development, poor energy levels, reduced endurance, poor sleep, problems with focus or concentration, and fatigue. These symptoms, in combination with visible difference in size compared to peers, lead to physical limitations impacting daily life, and social, or emotional impacts. The severity of GHD impact on children and adolescents appears to be variable and individualized, but these data indicate that early identification and treatment may lead to fewer impacts. There is also a need for further research in these aspects of care for children with GHD.

## References

[1] Wille N, Erhart M, Petersen C, Ravens-Sieberer U (2008). The impact of overweight and obesity on health-related quality of life in childhood–results from an intervention study. BMC Public Health.

[2] Zeller MH, Modi AC (2009). Development and initial validation of an obesity-specific quality-of-life measure for children: Sizing me up. Obesity (Silver Spring).

[3] Lillis J, Hayes SC, Bunting K, Masuda A (2009). Teaching acceptance and mindfulness to improve the lives of the obese: a preliminary test of a theoretical model. Annals of Behavioral Medicine: A Publication of the Society of Behavioral Medicine.

[4] Thomas M, Massa G, Craen M, de Zegher F, Bourguignon JP, Heinrichs C (2004). Prevalence and demographic features of childhood growth hormone deficiency in Belgium during the period 1986–2001. European Journal of Endocrinology/European Federation of Endocrine Societies.

[5] Vimpani GV, Vimpani AF, Lidgard GP, Cameron EH, Farquhar JW (1977). Prevalence of severe growth hormone deficiency. British Medical Journal.

[6] Lindsay R, Feldcamp M, Harris D, Robertson J, Rallison M (1994). Utah growth study: Growth standards and the prevalence of growth hormone deficiency. The Journal of Pediatrics.

[7] Stochholm K, Gravholt CH, Laursen T, Jørgensen JO, Laurberg P, Andersen M (2006). Incidence of GH deficiency—a nationwide study. European Journal of Endocrinology/European Federation of Endocrine Societies.

[8] Stabler B, Clopper RR, Siegel PT, Stoppani C, Compton PG, Underwood LE (1994). Academic achievement and psychological adjustment in short children. The national cooperative growth study. Journal of Developmental and Behavioral Pediatrics: JDBP.

[9] Chaplin JE, Kriström B, Jonsson B, Hägglöf B, Tuvemo T, Aronson AS (2001). Improvements in behaviour and self-esteem following growth hormone treatment in short prepubertal children. Hormone Research in Paediatrics.

[10] Matza LS, Patrick DL, Riley AW, Alexander JJ, Rajmil L, Pleil AM (2013). Pediatric patient-reported outcome instruments for research to support medical product labeling: Report of the ISPOR PRO good research practices for the assessment of children and adolescents task force. Value in Health: The Journal of the International Society for Pharmacoeconomics and Outcomes Research.

[11] Corbin, J., Strauss, A. (2008). *Basics of qualitative research: Grounded theory procedures and techniques* (3rd ed.). Thousand Oaks: Sage.

[12] Dedoose. Version 7.0.21. (2016). Manhattan Beach, CA: Sociocultural research consultants, LLC. http://www.dedoose.com.

[13] Geisler A, Lass N, Reinsch N, Uysal Y, Singer V, Ravens-Sieberer U (2012). Quality of life in children and adolescents with growth hormone deficiency: Association with growth hormone treatment. Hormone Research in Paediatrics.

[14] Chaplin JE, Kriström B, Jonsson B, Hägglöf B, Tuvemo T, Aronson AS (2011). Improvements in behaviour and self-esteem following growth hormone treatment in short prepubertal children. Hormone Research in Paediatrics.

[15] Jovinelly, J. (2016). Growth hormone deficiency. Healthline Networks, Inc. Retrieved on 9 Jan, 2014 from, http://www.healthline.com/health/growth-hormone-deficiency?toptoctest=expand.

